# Dehydration synthesis and crystal structure of terbium oxychloride, TbOCl

**DOI:** 10.1107/S2056989020004387

**Published:** 2020-04-03

**Authors:** Saehwa Chong, Brian J. Riley, Zayne J. Nelson

**Affiliations:** a Pacific Northwest National Laboratory, Richland, WA 99352, USA

**Keywords:** oxychloride, rare-earth oxyhalide, powder diffraction

## Abstract

Terbium oxychloride, TbOCl, was synthesized *via* the simple heat-treatment of TbCl_3_·6H_2_O and its structure was determined by refinement against X-ray powder diffraction data. TbOCl crystallizes with the matlockite (PbFCl) structure in the tetra­gonal space group *P*4/*nmm* and is composed of alternating (001) layers of (TbO)_*n*_ and *n* Cl^−^. The unit-cell parameters, unit-cell volume, and density were compared to the literature data of other isostructural rare-earth oxychlorides in the same space group and showed good agreement when compared to the calculated trendlines.

## Abstract 2

Terbium oxychloride, TbOCl, was synthesized *via* the simple heat-treatment of TbCl_3_·6H_2_O and its structure was determined by refinement against X-ray powder diffraction data. TbOCl crystallizes with the matlockite (PbFCl) structure in the tetra­gonal space group *P*4/*nmm* and is composed of alternating (001) layers of (TbO)_*n*_ and *n* Cl^−^.

## Chemical context   

Rare-earth oxychlorides, *RE*OCl, are promising materials for various applications including use as catalysts, sensors, and phosphors (Podkolzin *et al.*, 2007 ▸; Au *et al.*, 1997 ▸; Peringer *et al.*, 2009 ▸; Marsal *et al.*, 2005 ▸,; Kim *et al.*, 2019 ▸; Berdowski *et al.*, 1984 ▸; Imanaka *et al.*, 2001*a* ▸,*b* ▸; Okamoto *et al.*, 2002 ▸; Kim *et al.*, 2014 ▸). LaOCl is a stable catalyst for converting methane to methyl chloride (Podkolzin *et al.*, 2007 ▸) and can be used as a sensor material to detect CO_2_ and Cl_2_ gases (Marsal *et al.*, 2005 ▸; Imanaka *et al.*, 2001*b* ▸). The EuOCl catalyst showed high efficiency in converting ethyl­ene to vinyl chloride (Scharfe *et al.*, 2016 ▸). The luminescent properties of *RE*O*X* (*RE* = La, Eu; *X* = F, Cl, Br, I) can be controlled to emit a wide range of visible light from blue to red by changing the crystal symmetries and compositions (Kim *et al.*, 2014 ▸, 2019 ▸). As part of our studies in this area, we now describe the dehydration synthesis and structure of the title compound.

## Structural commentary   

**Figure 1:**
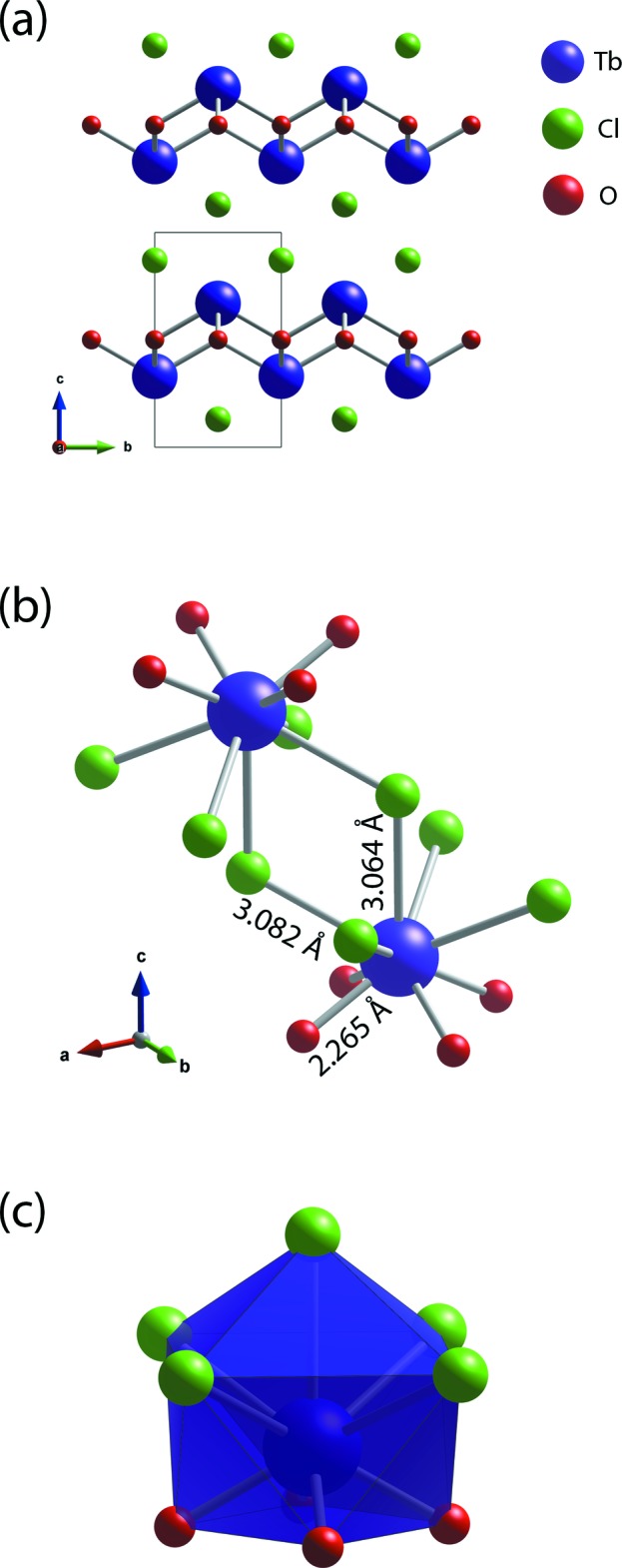
(*a*) Crystal structure of TbOCl, (*b*) the coordination environment of Tb, and (*c*) polyhedron representation of the Tb environment.

**Figure 2:**
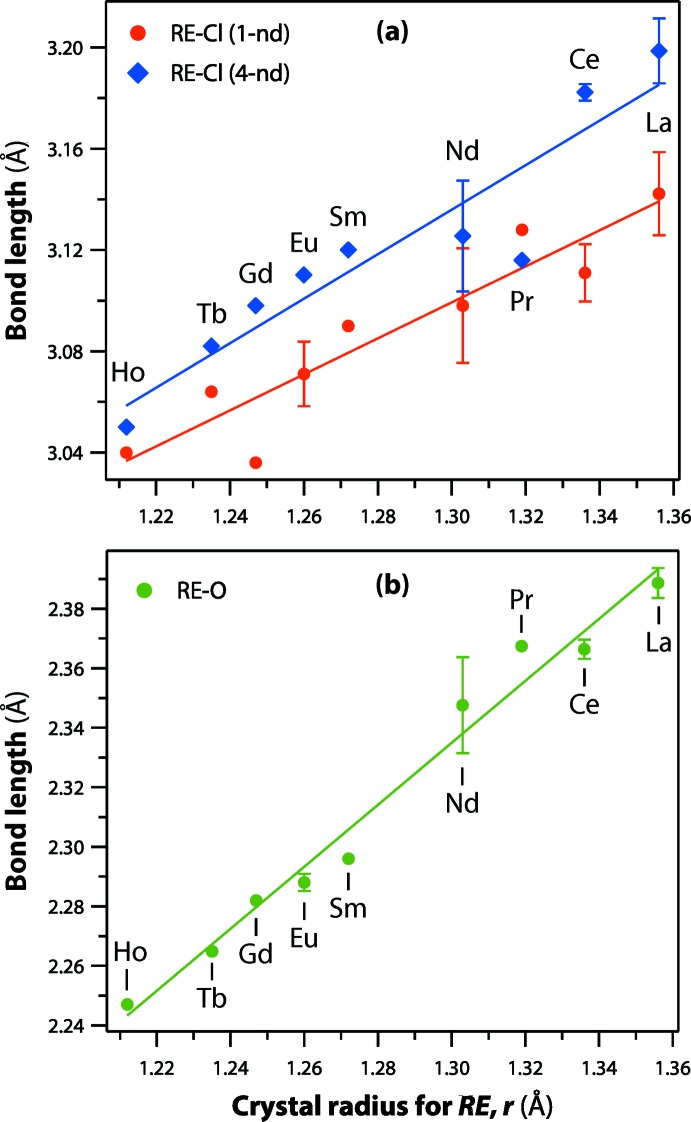
The *RE*—Cl and *RE*—O bond lengths in the *RE*OCl compounds listed in Table 1 ▸ as a function of *RE* crystal radius (coordination = 9) according to Shannon (1976 ▸). Where multiple values were available, averages and standard deviations are included for the datapoints. For (*a*), 1-nd and 4-nd denote 1 and 4 neighbor distances, respectively

The structural parameters of *RE*OCl (*RE* = La, Ce, Pr, Nd, Sm, Eu, Gd, Tb, Dy, Ho) in the literature and current study are summarized in Table 1 ▸. All these *RE*OCl compounds crystallize in the matlockite (PbFCl; Bannister, 1934 ▸) structure within the tetra­gonal *P*4/*nmm* space group. The crystal structure of TbOCl contains alternating (001) layers of (TbO)_*n*_ and *n* Cl^−^ (Fig. 1 ▸ *a*). The Tb cation is coordinated by five chloride ions and four oxygen atoms, forming a mono-capped TbO_4_Cl_5_ square anti­prism (Fig. 1 ▸ *b* and 1*c*). The *RE*—Cl and *RE*—O bond lengths in the *RE*OCl compounds are provided in Table 1 ▸. With larger *RE* cations in the structures, the *RE*—Cl and *RE*—O bond lengths increase (Fig. 2 ▸).

**Figure 3:**
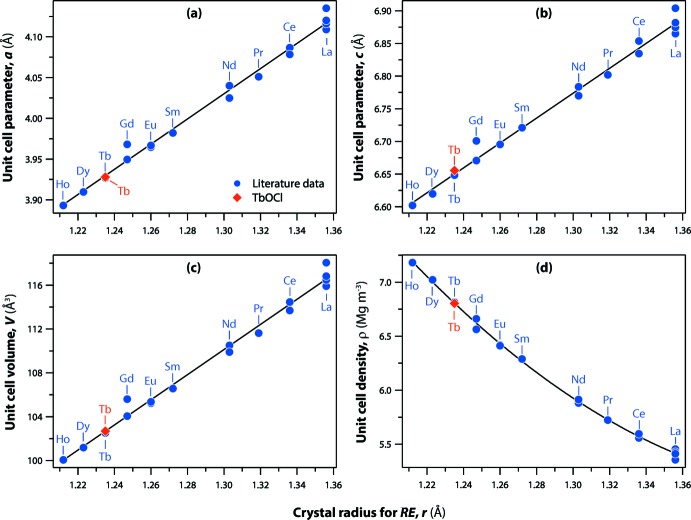
(*a*, *b*) Unit-cell parameters (*a* and *c*, respectively), (*c*) unit-cell volumes, and calculated unit-cell densities as a function of the crystal radius of the *RE* (coordination = 9) according to Shannon (1976 ▸) compared to literature values provided in Table 1 ▸.

The shortest Cl⋯Cl separation in TbOCl is 3.271 (4) Å, which compares with the van der Waals diameter of a Cl^−^ ion of about 3.62 Å. The Cl⋯Cl distances of other *RE*OCl compounds are also short, ranging from 3.24 to 3.46 Å on going from Ho^3+^ to La^3+^. With non-bonded vectors shorter than the van der Waals separation, strong inter­actions between atoms are expected in the structure (Maslen *et al.*, 1996 ▸). Templeton & Dauben (1953 ▸) mention the presence of weaker anion–anion repulsion between Cl atoms in *RE*OCl structures. The structural parameters of TbOCl were compared with the trendlines calculated using the values from Table 1 ▸ (Fig. 3 ▸). The unit-cell parameters and volumes increase linearly with the larger *RE* cations (Shannon, 1976 ▸) whereas the densities decrease non-linearly, fitting well to a 2nd order polynomial trend.

## Synthesis and crystallization   

The title compound was synthesized by a simple heat treatment of TbCl_3_·6H_2_O (Alfa Aesar, 99.99%). About 0.5 g of TbCl_3_·6H_2_O was placed in an alumina crucible, heated to 400°C at 5°C min^−1^, held for 8 h, and then cooled to room temperature at 5°C min^−1^. This synthesis method was used in our previous study (Riley *et al.*, 2018 ▸). The resulting product was a light-brown powder, which was ground in a mortar and pestle for X-ray powder diffraction analysis.

## Refinement   

**Figure 4:**
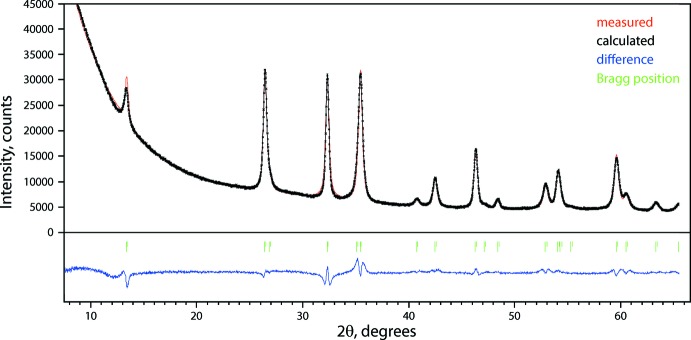
Measured, calculated, and difference XRD patterns of TbOCl.

Crystal data, data collection and structure refinement details are summarized in Table 2 ▸. The unit-cell parameters were obtained using *TOPAS* (version 4.2; Bruker, 2009 ▸) by refining the GdOCl pattern (ICSD 77820) with geometrical and chemical resemblance as a starting model. The Rietveld refinement was performed using *JANA2006* (Petříček *et al.*, 2014 ▸) with the obtained unit-cell parameters as initial values. A pseudo-Voigt function with other peak-shape parameters were used to fit peaks, and the background was modeled with a Chebychev polynomial. The plot of the Rietveld refinement result is shown in Fig. 4 ▸. The final refinement converged at *R*_wp_ = 3.22%.

## Supplementary Material

Crystal structure: contains datablock(s) global, I. DOI: 10.1107/S2056989020004387/hb7896sup1.cif

Rietveld powder data: contains datablock(s) I. DOI: 10.1107/S2056989020004387/hb7896Isup2.rtv

Structure factors: contains datablock(s) I. DOI: 10.1107/S2056989020004387/hb7896Isup3.hkl

CCDC reference: 1993793

Additional supporting information:  crystallographic information; 3D view; checkCIF report

## Figures and Tables

**Table 1:** Structural parameters of *RE*OCl compounds All compounds crystallize in the *P*4/*nmm* space group. For the *RE*—Cl bond lengths, the first value refers to one neighboring Cl atom, and the second number refers to four neighboring Cl atoms. Densities are calculated from crystallographic data.

*RE*	*a*(Å)	*c*(Å)	*V* (Å^3^)	Density(g cm^−3^)	*RE*—O(Å)	*RE*—Cl(Å)	Cl⋯Cl(Å)	Cl⋯O(Å)	O⋯O (Å)	ICSD/PDF
Ho	3.893	6.602	100.1	7.182	2.247	3.04, 3.05	3.24	3.12	2.753	76171 (Templeton & Dauben, 1953 ▸)
Dy	3.91	6.62	101.2	7.023						00–047-1725 (Kirik *et al.*, 1996 ▸)
Tb	3.9269	6.648	102.5	6.815						00–048-1648 (Kirik *et al.*, 1996 ▸)
Tb	3.9279	6.6556	102.7	6.804	2.2649	3.064, 3.082	3.271	3.151	2.7774	Current study
Gd	3.9495	6.6708	104.1	6.661	2.2839	3.036, 3.098	3.267	3.176	2.7927	59232 (Meyer & Schleid, 1986 ▸)
Gd	3.9698	6.7008	105.6	6.564	2.28	3.212, 3.071	3.428	3.089	2.8071	77820 (Hölsä *et al.*, 1996 ▸)
Eu	3.9646	6.695	105.2	6.42	2.286	3.08, 3.11	3.3	3.17	2.8034	28529 (Bärnighausen *et al.*, 1965 ▸)
Eu	3.9668	6.6955	105.4	6.412	2.2901	3.062, 3.1103	3.289	3.183	2.80492	54682 (Schnick, 2004 ▸)
Sm	3.982	6.721	106.6	6.289	2.296	3.09, 3.12	3.31	3.19	2.8157	26581 (Templeton & Dauben, 1953 ▸)
Nd	4.04	6.77	110.5	5.882	2.359	3.114, 3.11	3.428	3.165	2.86	31665 (Zachariasen, 1949 ▸)
Nd	4.0249	6.7837	109.9	5.914	2.3362	3.082, 3.141	3.343	3.221	2.84603	59231 (Meyer & Schleid, 1986 ▸)
Pr	4.053	6.799	111.7	5.723	2.3674	3.128, 3.116	3.441	3.178	2.866	31664 (Zachariasen, 1949 ▸)
Ce	4.0866	6.8538	114.5	5.558	2.3687	3.1190, 3.1846	3.3942	3.2572	2.8897	412069 (Schnick, 2004 ▸)
Ce	4.0785	6.8346	113.7	5.596	2.36413	3.103, 3.180	3.38	3.254	2.88393	72154 (Wołcyrz & Kepinski, 1992 ▸)
La	4.109	6.865	115.9	5.454	2.39	3.14, 3.18	3.45	3.24	2.9055	24611 (Sillen & Nylander, 1941 ▸)
La	4.117	6.881	116.6	5.42	2.3866	3.126, 3.2046	3.416	3.2751	2.9112	40297 (Brixner & Moore, 1983 ▸)
La	4.1351	6.904	118.1	5.355	2.395	3.165, 3.209	3.457	3.268	2.92397	77815 (Hölsä *et al.*, 1996 ▸)
La	4.1162	6.8746	116.5	5.428	2.3832	3.138, 3.201	3.425	3.265	2.9106	84330 (Hölsä *et al.*, 1997 ▸)
La	4.12	6.882	116.8	5.412						00–008-0477 (Swanson *et al.*, 1957 ▸)

**Table 2:** Experimental details

Crystal data	
Chemical formula	TbOCl
*M*_r_	210.4
Crystal system, space group	Tetragonal, *P*4/*n* *m* *m*
Temperature (K)	293
*a*, *c* (Å)	3.9279 (2), 6.6556 (5)
*V* (Å^3^)	102.68 (1)
*Z*	2
Radiation type	Cu *K*α, λ = 1.54188 Å
Specimen shape, size (mm)	Cylinder, 25 × 25

Data collection	
Diffractometer	Bruker D8 Advance
Specimen mounting	Packed powder pellet
Data collection mode	Reflection
Scan method	Step
2θ values (°)	2θ_min_ = 5, 2θ_max_ = 68.977, 2θ_step_ = 0.019

Refinement	
*R* factors and goodness of fit	*R*_p_ = 0.020, *R*_wp_ = 0.032, *R*_exp_ = 0.009, *R*(*F*) = 0.033, χ^2^ = 13.690
No. of parameters	17

Computer programs: *XRD Commander* (Kienle & Jacob, 2003 ▸), *TOPAS* (Bruker, 2009 ▸), *SUPERFLIP* (Palatinus & Chapuis, 2007 ▸), *JANA2006* (Petříček *et al.*, 2014 ▸), *VESTA* (Momma & Izumi, 2011 ▸) and *publCIF* (Westrip, 2010 ▸).

## supplementary crystallographic information

### Crystal data

**Table:**

TbOCl	*Z* = 2
*M**_r_* = 210.4	*D*_x_ = 6.804 Mg m^−^^3^
Tetragonal, *P*4/*n**m**m*	Cu *K*α radiation, λ = 1.54188 Å
*a* = 3.9279 (2) Å	*T* = 293 K
*c* = 6.6556 (5) Å	light brown
*V* = 102.68 (1) Å^3^	cylinder, 25 × 25 mm

### Data collection

**Table:**

Bruker D8 Advance diffractometer	Data collection mode: reflection
Radiation source: sealed X-ray tube	Scan method: step
Specimen mounting: packed powder pellet	2θ_min_ = 5°, 2θ_max_ = 68.977°, 2θ_step_ = 0.019°

### Refinement

**Table:**

*R*_p_ = 0.020	17 parameters
*R*_wp_ = 0.032	Weighting scheme based on measured s.u.'s
*R*_exp_ = 0.009	(Δ/σ)_max_ = 0.030
*R*(*F*) = 0.033	Background function: 8 Chebyshev polynoms
3292 data points	Preferred orientation correction: March & Dollase
Profile function: Pseudo-Voigt	

### Fractional atomic coordinates and isotropic or equivalent isotropic displacement parameters (Å^2^)

**Table:**

	*x*	*y*	*z*	*U*_iso_*/*U*_eq_	
Tb1	0.5	0	0.3305 (2)	0.002	
Cl1	0	0.5	0.1298 (9)	0.002	
O1	1	0	0.5	0.002	

### Atomic displacement parameters (Å^2^)

**Table:**

	*U*^11^	*U*^22^	*U*^33^	*U*^12^	*U*^13^	*U*^23^
Tb1	0.002	0.002	0.002	0	0	0
Cl1	0.002	0.002	0.002	0	0	0
O1	0.002	0.002	0.002	0	0	0

### Geometric parameters (Å, º)

**Table:**

Tb1—Tb1^i^	3.5784 (13)	Tb1—O1^v^	2.2649 (7)
Tb1—Tb1^ii^	3.5784 (13)	Tb1—O1	2.2649 (7)
Tb1—Tb1^iii^	3.5784 (13)	Tb1—O1^vi^	2.2649 (7)
Tb1—Tb1^iv^	3.5784 (13)	Tb1—O1^vii^	2.2649 (7)
			
Tb1^i^—Tb1—Tb1^ii^	66.57 (2)	Tb1^iii^—Tb1—O1^vii^	99.31 (4)
Tb1^i^—Tb1—Tb1^iii^	66.57 (2)	Tb1^iv^—Tb1—O1^v^	99.31 (4)
Tb1^i^—Tb1—Tb1^iv^	101.82 (4)	Tb1^iv^—Tb1—O1	37.817 (14)
Tb1^i^—Tb1—O1^v^	37.817 (14)	Tb1^iv^—Tb1—O1^vi^	99.31 (4)
Tb1^i^—Tb1—O1	99.31 (4)	Tb1^iv^—Tb1—O1^vii^	37.817 (14)
Tb1^i^—Tb1—O1^vi^	37.817 (14)	O1^v^—Tb1—O1	120.25 (6)
Tb1^i^—Tb1—O1^vii^	99.31 (4)	O1^v^—Tb1—O1^vi^	75.63 (3)
Tb1^ii^—Tb1—Tb1^iii^	101.82 (4)	O1^v^—Tb1—O1^vii^	75.63 (3)
Tb1^ii^—Tb1—Tb1^iv^	66.57 (2)	O1—Tb1—O1^vi^	75.63 (3)
Tb1^ii^—Tb1—O1^v^	37.817 (14)	O1—Tb1—O1^vii^	75.63 (3)
Tb1^ii^—Tb1—O1	99.31 (4)	O1^vi^—Tb1—O1^vii^	120.25 (6)
Tb1^ii^—Tb1—O1^vi^	99.31 (4)	Tb1—O1—Tb1^viii^	120.25 (4)
Tb1^ii^—Tb1—O1^vii^	37.817 (14)	Tb1—O1—Tb1^iii^	104.37 (2)
Tb1^iii^—Tb1—Tb1^iv^	66.57 (2)	Tb1—O1—Tb1^iv^	104.37 (2)
Tb1^iii^—Tb1—O1^v^	99.31 (4)	Tb1^viii^—O1—Tb1^iii^	104.37 (2)
Tb1^iii^—Tb1—O1	37.817 (14)	Tb1^viii^—O1—Tb1^iv^	104.37 (2)
Tb1^iii^—Tb1—O1^vi^	37.817 (14)	Tb1^iii^—O1—Tb1^iv^	120.25 (4)

Symmetry codes: (i) −*x*+1/2, *y*−1/2, −*z*+1; (ii) −*x*+1/2, *y*+1/2, −*z*+1; (iii) −*x*+3/2, *y*−1/2, −*z*+1; (iv) −*x*+3/2, *y*+1/2, −*z*+1; (v) *x*−1, *y*, *z*; (vi) −*y*+1/2, *x*−3/2, *z*; (vii) −*y*+1/2, *x*−1/2, *z*; (viii) *x*+1, *y*, *z*.
